# Analysis of linear and non-linear genotype × environment interaction

**DOI:** 10.3389/fgene.2014.00227

**Published:** 2014-07-22

**Authors:** Rong-Cai Yang

**Affiliations:** ^1^Alberta Agriculture and Rural DevelopmentEdmonton, AB, Canada; ^2^Department of Agricultural, Food and Nutritional Science, University of AlbertaEdmonton, AB, Canada

**Keywords:** barley, environmental index, estimation, genotype × environment interaction, non-linear functions, quantitative trait loci

## Abstract

The usual analysis of genotype × environment interaction (G × E) is based on the linear regression of genotypic performance on environmental changes (e.g., classic stability analysis). This linear model may often lead to lumping together of the non-linear responses to the whole range of environmental changes from suboptimal and super optimal conditions, thereby lowering the power of detecting G × E variation. On the other hand, the G × E is present when the magnitude of the genetic effect differs across the range of environmental conditions regardless of whether the response to environmental changes is linear or non-linear. The objectives of this study are: (i) explore the use of four commonly used non-linear functions (logistic, parabola, normal and Cauchy functions) for modeling non-linear genotypic responses to environmental changes and (ii) to investigate the difference in the magnitude of estimated genetic effects under different environmental conditions. The use of non-linear functions was illustrated through the analysis of one data set taken from barley cultivar trials in Alberta, Canada (Data A) and the examination of change in effect sizes is through the analysis another data set taken from the North America Barley Genome Mapping Project (Data B). The analysis of Data A showed that the Cauchy function captured an average of >40% of total G × E variation whereas the logistic function captured less G × E variation than the linear function. The analysis of Data B showed that genotypic responses were largely linear and that strong QTL × environment interaction existed as the positions, sizes and directions of QTL detected differed in poor vs. good environments. We conclude that (i) the non-linear functions should be considered when analyzing multi-environmental trials with a wide range of environmental variation and (ii) QTL × environment interaction can arise from the difference in effect sizes across environments.

## Introduction

Inconsistent performance of genotypes over different environments known as genotype × environment interaction (G × E) remains to be a major impediment to genetic improvement of biological species in Canada and elsewhere. G × E is particularly important for plant species (e.g., agricultural crops and forest trees) because they spend their entire life at the same locality. Over the past decades, the assessment of G × E has been done with the data obtained from testing of the same genotypes over multiple environments (locations or years), i.e., multi-environmental trials (Yang, 2007).

The G × E effect has been incorporated into quantitative genetic models (Falconer and Mackay, 1996) through the use of genetic correlations within and between individual genotypes (e.g., Crossa et al., 2004; Burgueño et al., 2008). The basic idea behind such an approach is to predict genetic values through borrowing information among individuals from genetic relationships, and within individuals (across environments) from genetic and environmental correlations. The analysis of such correlation structure has been performed to obtain the parsimony description of G × E variation using different versions of linear-bilinear models based on a mathematical technique known as singular value decomposition (SVD) (Golub and Reinsch, 1970). One popular use of the SVD technique is the biplot analysis of G × E based on the two commonly used rank-two linear-bilinear models: the additive main effects and multiplicative interaction (AMMI) model and the genotype main effects and genotype × environment interaction effects (GGE) model (i.e., fitted to residuals after removal of environment main effects) (for review, see Yang et al., 2009). Recently, Burgueño et al. (2008) and Cullis et al. (2010) described a similar biplot analysis under a mixed-model framework using a series of rank-two factor-analytic (FA) model. Apart from the adequacy of the rank-two models and other statistical issues, Yang et al. (2009) pointed out that the biplot analysis has contributed little to our understanding of the nature of G × E variation because it is a descriptive analysis with little predictive power.

Baker (1988) and others (e.g., Scheiner, 1993; Lindgren and Ying, 2000) have suggested the use of predictive models based on linear and non-linear response functions for studying G × E. The classic stability analysis based on simple linear regression model as pioneered by Yates and Cochran (1938) is a special case of the general non-linear predictive models. In addition, linear functions would usually account for a small portion of G × E variation if a wide range of environmental conditions are tested. On the other hand, for quantitative traits such as crop yield or human complex diseases (Franks et al., 2013), the G × E is manifested when the magnitude of the genetic effect differs across the range of environmental conditions regardless of whether the response to environmental changes is linear or non-linear. For this reason, many recent genome-wide association studies (GWAS) in human (Kilpelainen et al., 2011; Qi et al., 2012) have focused on determining the effect sizes of causal variants (e.g., SNPs) over different environmental conditions (e.g., different lifestyle behaviors).

The objectives of this paper are two folds. First, we investigate the use of different non-linear functions for modeling genotypic response to environmental changes or gradients. In this case, G × E is present when the response curves fail to be parallel (Baker, 1988). Similar concept has been used in evolution and ecology but under different names [e.g., phenotypic plasticity (robustness), reaction norm] (e.g., Via et al., 1995). Second, we examine whether there are differences in estimated genetic effects under different environmental conditions. It is generally expected that a larger effect is more likely found in the environmental condition where the expression of a gene is facilitated than in the environmental condition where the expression of a gene is not facilitated.

## Materials and methods

### Description of non-linear functions

As a starting point, we provide a brief description of the classic stability analysis that is based on a linear regression function (Yates and Cochran, 1938; Finlay and Wilkinson, 1963; Eberhart and Russell, 1966; Perkins and Jinks, 1968):

(1) $$y_{ij}=a_{i}+b_{i}x_{j}$$

Where *y*_*ij*_ is the performance (say yield) of the *i*th genotype tested in *j*th environment, *x*_*j*_ is the mean yield of all genotypes tested in the *j*th environment (known as environmental index), the intercept *a*_*i*_ is the yield of the *i*th genotype at the worst environment, and the slope *b*_*i*_ measures the stability of the *i*th genotype.

According to Finlay and Wilkinson (1963), all genotypes can be conveniently classified into three groups: (i) genotypes with average stability (*b*_*i*_ = 1.0); (ii) genotypes with low stability or high sensitivity to environmental changes (*b*_*i*_ > 1.0) and (iii) genotypes with high stability or low sensitivity to environmental changes (*b*_*i*_ < 1.0). Eberhart and Russell (1966) further refined this definition by suggesting that a stable genotype would be the one with average stability, low variance due to deviations from regression and high mean yield.

However, linear response usually accounts for only a small portion of the G × E variation and the responses are most often non-linear in practice (Knight, 1973; Jinks and Pooni, 1988). This occurs because when individuals of the same genotype are evaluated at different levels of an environmental factor ranging from suboptimal, optimal to super-optimal levels, their performance (i.e., yield) often shows a continuous non-linear relationship with the environment. The response curve can rise from near zero performance at extreme suboptimal levels of the environmental factor to some asymptotic value at optimal levels, and then decrease to near zero value at extreme super-optimal levels. If a small portion of the environmental range is evaluated, only the linear response could possibly be observed within this limited range of environmental conditions.

Here we briefly describe some well-known non-linear functions that have been used to model relationships of yield or growth with a single more defined environmental variable (for details, see Baker, 1988; Ratkowsky, 1993). The most obvious non-linear function is a quadratic function (parabola function) and it is often used to describe the relationship between grain yield and field water availability (e.g., McKenzie et al., 2004):

(2) $$y_{ij}=a_{i}+b_{i}x_{j}+c_{i}x_{j}^{\;2}$$

The quadratic function has been also used to describe the genetic response to climate variables in forest trees (Rehfeldt et al., 1999). Another non-linear function is the reciprocal of the quadratic function used to describe the relationship between yield and planting density (Baker, 1988):

(3) $$y_{ij}^{-1}=a_{i}+b_{i}x_{j}+c_{i}x_{j}^{\;2}$$

This general expression can take several special forms, one of which is known as Cauchy function,

(4) $$y_{ij}=\frac{k_{i}}{\left[1+\frac{{\left(x_{j}-x_{\max}\right)}^{2}}{r_{i}^{2}}\right]}$$

Where *K*_*i*_ is a parameter that scales yield from zero to one (i.e., 0 ≤ *K*_*i*_ ≤ 1), *x*_max_ is the *x* value at which the maximum yield is achieved and γ_*i*_ is the scale parameter which measures the range of genotypic response to environmental changes. This Cauchy function has been used to delineate breeding zones in forest trees (Raymond and Lindgren, 1990; Lindgren and Ying, 2000). The logistic curve:

(5) $$y_{ij}^{-1}=a_{i}+b_{i}c_{j}^{x_{j}}$$

is often used to describe the plant growth with age, but it can also be useful for the response to the environmental changes (Baker, 1988; West et al., 2001; Zuo et al., 2012). Roberds and Namkoong (1989) proposed the use of the Gaussian function to model the genotypic response to an environmental gradient:

(6) $$y_{ij}=\frac{k_{i}}{\sqrt{2\pi r_{i}^{2}}}e^{\left[\frac{{\left(x_{j}-x_{\max}\right)}^{2}}{2r_{i}^{2}}\right]}$$

**Figure 1:**
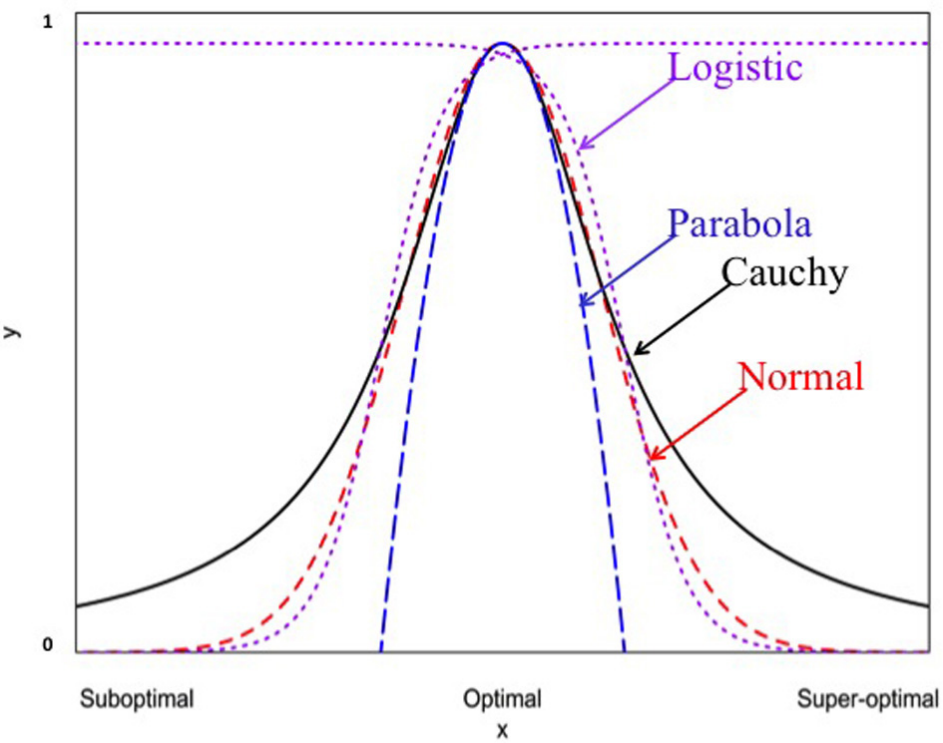
**Four different non-linear functions for studying genotype-environment interaction (normal, Cauchy, parabola, and logistic)**.

When *K*_*i*_ = 1, Equation (6) becomes the normal probability density function. These non-linear functions are graphed in Figure 1. It should be noted that the y-axis and x-axis in Figure 1 are rescaled in standardized units. For example, the standardized Cauchy function is given by:

(7) $${y^{\prime }}_{ij}=\frac{1}{1+{x^{\prime }}_{ij}^{2}}$$

Where ${y^{\prime }}_{ij}=\frac{y_{ij}}{k_{i}}$ and ${x^{\prime }}_{ij}=\frac{x_{j}-x_{\text{max}}}{r_{i}}$ Thus, *y*′_*ij*_ becomes a relative measure of the performance within the range of 0 (0%)–1 (100%). All non-linear functions are indistinguishable at or near the optimum *x*′_*ij*_ = 0. For example, the Cauchy function can be well approximated by a quadratic function at the rescaled axises because of the following mathematical relationship:

(8) $$\frac{1}{1+{x^{\prime }}_{ij}^{2}}\to 1-{x^{\prime }}_{ij}^{2}\text{when}x_{ij^{\prime }}\to 0$$

but the approximation becomes less desirable at the extreme environmental conditions (i.e., |*x*′_*ij*_| >> 0).

### Analysis of empirical data

We will describe the analysis of two empirical data sets. The first data set (Data A) is taken from Yang et al. (2006) who analyzed 324 replicated barley cultivar trials sown at 84 sites across three provinces (Alberta, Saskatchewan and Manitoba) in the Canadian prairies during 1995–2003. Here we illustrate the use of non-linear G × E analysis of the data taken from the trials in the province of Alberta only. The data set for the analysis is briefly recapitulated now. In each year, there were 16 (1995)–22 (2000) trials planted at different locations across Alberta. Each trial consisted of 39–44 barley cultivars. It should be pointed that in a given year, the same cultivars were usually included in each and every trial but over different years, at least some cultivars were different in the same and different test sites either due to a turnover to newly registered cultivars or to unavailability of pedigree seed of older cultivars. The same check cultivars were used across the different years. All trials were conducted using a randomized complete block design with three or four replications. Cultural practices such as fertility, tillage and pest control varied from site to site but were considered to be the most appropriate for the individual sites.

Following the procedure of Yang et al. (2006), the usual analysis of variance partitioned the total sum of squares in each year into components due to the site effects (E), the cultivar effects (G) and the interaction between cultivar and site effects (G × E) using SAS PROC MIXED (Sas Institute Inc, 2012). Further partitioning of the G × E variation under different non-linear functions was carried out using appropriate data transformations that enabled the analysis of non-linear G × E under the mixed-model framework. The different non-linear functions were compared interms of their ability to capture the amount of G × E variation.

The second data set (Data B) is a publicly available data set that we previously analyzed using single-marker analysis (Ham et al., 2010) and genome-wide prediction (Yang and Ham, 2012). The data set consisted of 150 doubled haploid (DH) lines that were developed from a cross between two malting barley varieties (Steptoe × Morex) for the North American Barley Genome Mapping Project (NABGMP) (http://wheat.pw.usda.gov). These DH lines were tested in 16 environments over North America for yield and seven other agronomic and malt quality traits. A total of 223 restricted fragment length polymorphism (RFLP) makers mapped over the seven chromosomes of the barley genome with 37, 37, 31, 33, 29, 22, and 34 makers being mapped on chromosomes 1, 2, 3, 4, 5, 6, and 7, respectively. The effects of these RFLP markers were estimated using a R package, GLMNET/R, at three representative environments: poor (minimum environmental index), average (mean environmental index) and good (maximum environmental index) environments. GLMNET/R implemented an efficient procedure for fitting the entire elastic-net regularization path for super-saturated linear regression as in genome-wide association studies (GWAS) (Friedman et al., 2010; R Core Team, 2012). The elastic-net penalty (P_α_) is a compromise between the ridge-regression penalty (α = 0) and the LASSO penalty (α = 1), where α is related to the degree of shrinkage of marker effects. Two shrinkage methods, elastic net with α = 0.5 and α = 1 (i.e., LASSO), were used for genome-wide estimation of marker effects on response at poor, average and good environments.

## Results

### Data A

We (Yang et al., 2006) previously partitioned the total variability into components due to genotypes (G), environments (E) and G × E, and G × E accounted for 6.6% (2003)–23.9% (2000) of the total variability across different years. Here we further partitioned the G × E variability into a component that could be explained by different linear and non-linear models described above and a residual (Table 1). This further partitioning was based on linear or non-linear regression of yield on the environmental index (calculated as the mean of all cultivars at each and every test location). It is evident from Table 1 that different non-linear models captured different amounts of the total G × E variation, ranging from an average of 10.2% for logistic model to 40.3% for Cauchy model. It is somewhat surprising that some non-linear models (e.g., logistic model) actually captured less G × E variation than the linear model. For a given model, there was also a large amount of year-to-year variation in the percentages of the G × E variation being captured. For example, Cauchy model captured 12.5% in 1997 and 86.5% in 2001. This result suggests that G × E variation is more predictable in some “good” years than in other “poor” years. In good years, stable and non-extreme weather or other agroclimatic conditions are available for optimal performance of individual genotypes whereas in poor years, such conditions do not exist.

**Table 1:** **Percentages of genotype × environment interaction variation explained by linear function and four non-linear functions in barley cultivar trials in Alberta tested in 1995–2003**.

**Year**	**Linear**	**Logistic**	**Parabola**	**Normal**	**Cauchy**
1995	8.49	7.52	11.10	11.47	20.17
1996	8.84	7.32	14.14	13.06	25.28
1997	6.72	5.88	11.81	9.97	12.54
1998	8.40	7.70	13.15	15.12	26.54
1999	14.70	15.75	20.41	20.85	36.56
2000	5.91	8.34	8.67	14.30	32.39
2001	6.95	11.77	13.16	35.04	86.45
2002	23.60	13.17	40.08	33.46	84.87
2003	17.71	14.06	22.51	18.88	37.69
Average	11.26	10.17	17.23	19.13	40.28

### Data B

**Figure 2:**
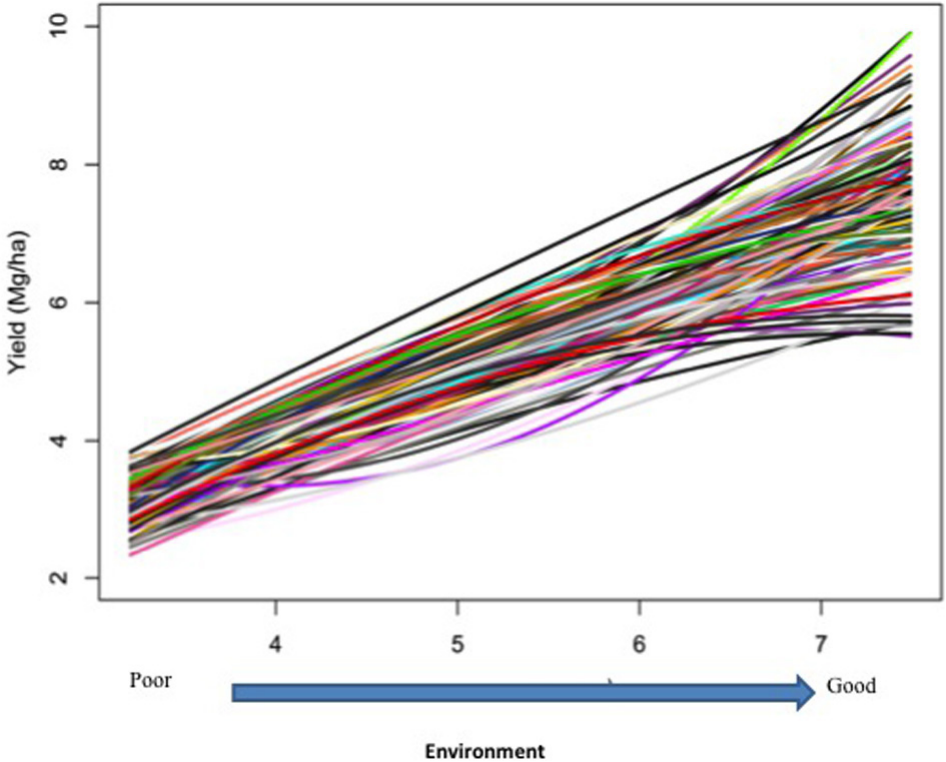
**Responses of 150 doubled-haploid lines of barley from a cross between two malting barley cultivars (Steptoe × Morex) for the North American Barley Genome Mapping Project (NABGMP)**. The range of the environmental index values runs from low (poor environment) to high (good environment).

**Figure 3:**
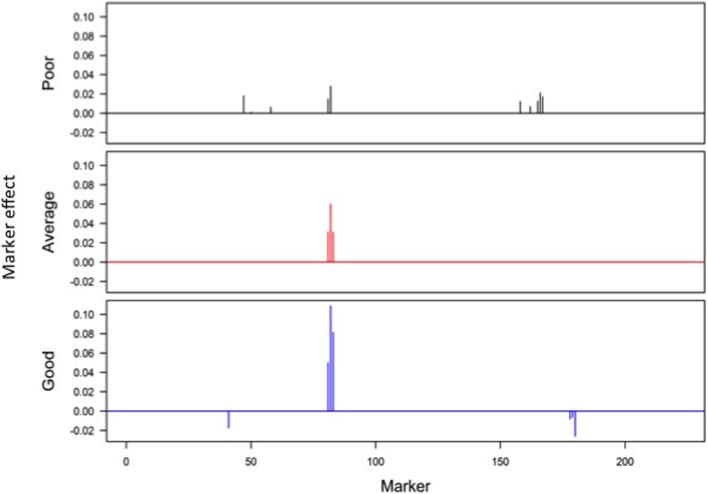
**Genome-wide scan of QTLs responsible for barley yield in poor, average, and good environments using the ridge regression analysis**.

**Figure 4:**
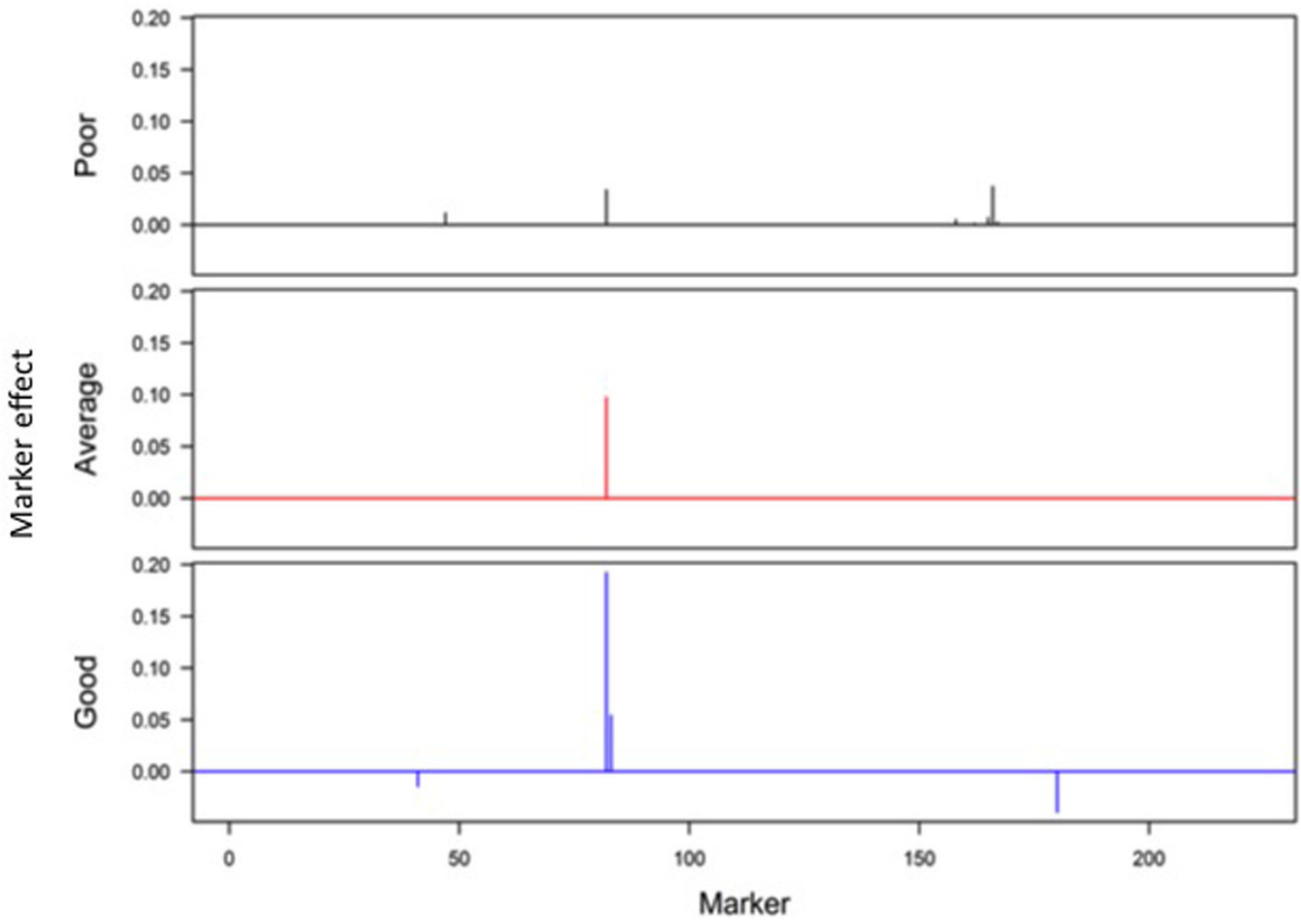
**Genome-wide scan of QTLs responsible for barley yield in poor, average, and good environments using the LASSO analysis**.

Responses of the DH lines to environmental index were examined under different linear and non-linear models. The responses of most DH lines were linear (Figure 2). Furthermore, the variation in such linear response was greater in “good” environments (i.e., the locations with higher environmental index values) than in “poor” environments (i.e., the locations with lower environmental index values). It is evident from Figures 3, 4 that Elastic net (α = 0.5) detected more marker effects than LASSO (α = 1.0) but LASSO gave much sharper resolution of marker effects. Under both estimation methods, marker effects were more pronounced in good environment than in poor environment.

## Discussion

**Figure 5:**
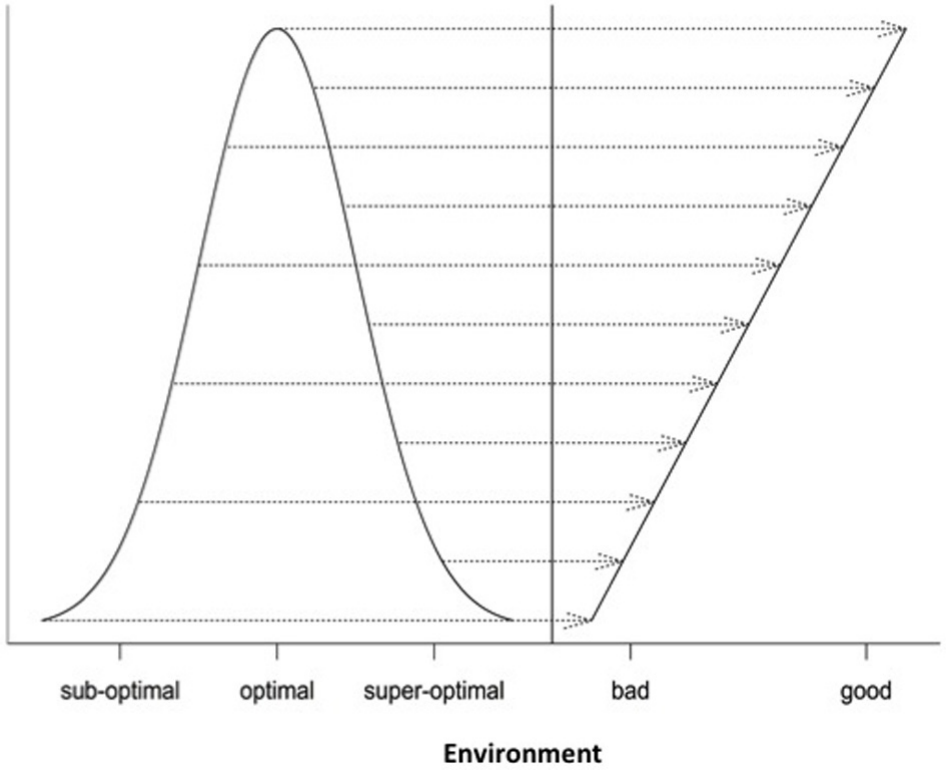
**A demonstration of masking true (non-linear) responses to environmental changes if a linear function is used**.

Differential responses of genotypes to environmental conditions (G × E interactions) can be linear or non-linear. Most current analyses of such responses are limited to the use of linear models. In this study, we explore the use of different non-linear models for characterizing and dissecting G × E interaction. This was done by extending the linear regression on environmental indexes (the means of all genotypic values at individual environments) or the classic stability analysis (Yates and Cochran, 1938; Finlay and Wilkinson, 1963; Eberhart and Russell, 1966; Perkins and Jinks, 1968) to the non-linear regression analysis. In the past, several non-linear functions including logistic, quadratic (parabola), Cauchy and normal functions have been individually used to describe genotypic responses to environments (e.g., Knight, 1973; Jinks and Pooni, 1979; Roberds and Namkoong, 1989; Raymond and Lindgren, 1990; Van Tienderen and Koelewijn, 1994; Lindgren and Ying, 2000). For example, Van Tienderen and Koelewijn (1994) found that the quadratic function was “statistically significantly better” than the linear function. In this study, our comparison of these representative non-linear functions (Figure 1) reveals the following characteristics. First of all, when the parameters are appropriately chosen or rescaled, the response curves of different non-linear functions near the optimum are indistinguishably similar, but their differences become increasingly evident when the environmental condition is not good (suboptimal) or too good (super-optimal). Second, should the true response be non-linear but be treated as linear, it would be difficult to tell the difference between non-linear responses to suboptimal and super-optimal conditions because in the linear analysis, both suboptimal and super-optimal conditions are lumped together to represent a deteriorated environment (Figure 5). Thus, the linear analysis would cause the reduced range of environmental variation when non-linear response is present but its presence unknown to the researcher or simply ignored! Third, including responses to both suboptimal and super-optimal conditions provides more opportunities to characterize the nature of G × E interaction. For example, differences in the rate of increase in response at suboptimal levels would reflect differences in efficiency but differences in the rate of decrease in response at super-optimal levels would reflect differences in tolerance.

It may not totally surprising from this study that the Cauchy function is the best in capturing the G × E variation because it may be best representative of how different genotype respond to the whole range of environmental conditions. Each genotype would have its own optimal growing environment. Any deviation from such optimum, either super-optimal or sub-optimal conditions, would cause a reduced performance or adaptation. The reduction must be very gentle for relatively mild super-optimal or sub-optimal conditions. For the extremely poor environments, the reduction asymptotically approaches a nonzero minimum. This scenario is best described by the Cauchy function which has a gentle decline at the regions close to the optimum (the center) and it has very long, flat tails at either side of the center but never converges. Comparing to the other non-linear functions, the Cauchy function is more sensitive to the values close to the optimum but less sensitive to the values at extreme environments which are of little practical interest (Raymond and Lindgren, 1990; Lindgren and Ying, 2000). Thus the Cauchy should be considered in future plant and animal breeding and evolution studies.

Our analysis of Data A shows that different non-linear functions captured different amounts of G × E interaction variation with Cauchy function capturing an average of 40% of the total G × E variation which is twice the amount captured by the second best model (normal function). This striking capability of Cauchy function was also observed in Raymond and Lindgren (1990) and Lindgren and Ying (2000). It is evident from Figure 1 that all non-linear functions are similar and indistinguishable when environmental conditions are close to the optimum but they become markedly different when environmental conditions move toward the extremes. Our results suggest that the actual range of environmental conditions as represented by all test locations over the years is too extended to be accommodated by all the functions except for the Cauchy function which can accommodate the environmental conditions at some distance away from the optimum. Thus, in practical applications, the choice of a non-linear function should be done after examining the actual distributions of environmental conditions either from previous experiences or from empirical data. It should also be reminded that a sufficient number of environments (e.g., ~40 locations in our study) are needed so that the true distribution of environmental conditions can be well approximated by the empirical data.

The results from the analysis of Data B reveal that responses of 150 DH lines to environmental indexes were largely linear (Figure 2). The 16 environments (essentially 12 locations in 2 years) at which these DH lines were tested would hardly be considered sufficient for covering the whole environmental range. Thus, the linear responses may be reflective of the response to a limited range of environmental indexes. The possibility of non-linear responses could not be ruled out particularly if the whole environmental range is available. Even within this limited environmental range, our analysis revealed some interconnected and interesting features. First of all, the variation in the responses of DH lines was greater in good environment than in poor environment. Second, the contrast between good and poor environments correspondingly led to the difference in the estimated positions, sizes and directions of QTL effects between these environments and this occurred irrespective of which method was used (Figures 3, 4). Third, inconsistency in the positions, sizes and directions of QTLs across the environmental range is a direct evidence of strong QTL × environment interaction.

As just mentioned above, there is increase in the effect size of detected QTLs in good environment in comparison to poor environment (Figures 3, 4). Similar observations have recently been made in many human GWAS particularly with respect to GWAS-discovered causal SNPs controlling the susceptibility of obesity. For example, Kilpelainen et al. (2011) showed that the risk effect of FTO (fat mass and obesity associated) alleles was about 100% and larger in physically inactive individuals than in active individuals from North America. Similar increase in the effect size was observed when individuals with ≥1 serving sugar-sweetened beverage per day were compared to those with sugary beverage intake <1 serving per month (Qi et al., 2012). Such increase in the effect size occurs because there are causal variants that lead to more phenotypic variation in the inactive lifestyle than in the active lifestyle. While generally being ignored in the past, our study and those other recent studies raise an important point that the genetic effects must not only be defined and estimated under a reference population, but also under an appropriate environment.

In conclusion, this paper calls for the attention to the use of non-linear functions for studying G × E interaction. We illustrate that the portion of G × E variation due to non-linear responses can be substantial if the correct non-linear function is used. We also emphasize that the correct identification of non-linear functions depends critically on how close the estimated environmental range is to the true range.

### Conflict of interest statement

The author declares that the research was conducted in the absence of any commercial or financial relationships that could be construed as a potential conflict of interest.
